# Silencing dentate newborn neurons alters excitatory/inhibitory balance and impairs behavioral inhibition and flexibility

**DOI:** 10.1126/sciadv.adk4741

**Published:** 2024-01-10

**Authors:** Haowei Li, Risako Tamura, Daiki Hayashi, Hirotaka Asai, Junya Koga, Shota Ando, Sayumi Yokota, Jun Kaneko, Keisuke Sakurai, Akira Sumiyoshi, Tadashi Yamamoto, Keigo Hikishima, Kazumasa Z. Tanaka, Thomas J. McHugh, Tatsuhiro Hisatsune

**Affiliations:** ^1^ Department of Integrated Biosciences, Graduate School of Frontier Sciences, The University of Tokyo, Kashiwa, Japan.; ^2^ Department of Molecular Imaging and Theranostics, National Institutes for Quantum and Radiological Science and Technology, Chiba, Japan.; ^3^ Okinawa Institute of Science and Technology Graduate University, Okinawa, Japan.; ^4^ Laboratory for Circuit and Behavioral Physiology, RIKEN Center for Brain Science, Saitama, Japan.

## Abstract

Adult neurogenesis confers the hippocampus with unparalleled neural plasticity, essential for intricate cognitive functions. The specific influence of sparse newborn neurons (NBNs) in modulating neural activities and subsequently steering behavior, however, remains obscure. Using an engineered NBN–tetanus toxin mouse model (NBN-TeTX), we noninvasively silenced NBNs, elucidating their crucial role in impulse inhibition and cognitive flexibility as evidenced through Morris water maze reversal learning and Go/Nogo task in operant learning. Task-based functional MRI (tb-fMRI) paired with operant learning revealed dorsal hippocampal hyperactivation during the Nogo task in male NBN-TeTX mice, suggesting that hippocampal hyperexcitability might underlie the observed behavioral deficits. Additionally, resting-state fMRI (rs-fMRI) exhibited enhanced functional connectivity between the dorsal and ventral dentate gyrus following NBN silencing. Further investigations into the activities of PV^+^ interneurons and mossy cells highlighted the indispensability of NBNs in maintaining the hippocampal excitation/inhibition balance. Our findings emphasize that the neural plasticity driven by NBNs extensively modulates the hippocampus, sculpting inhibitory control and cognitive flexibility.

## Teaser

Noninvasive silencing of newborn neurons unveils learning deficits and detrimental hippocampal activation.

## INTRODUCTION

Organized memories and controlled forgetting help us navigate complex environments, preventing cognitive overload (*1*, *2*). This balance largely hinges on executive functions, predominantly comprising working memory, inhibitory control, and cognitive flexibility (*3**–**5*). Among these, cognitive flexibility necessitates not only intact working memory but also adept impulse inhibition. This proficiency ensures that individuals can judiciously delay or suppress maladaptive behaviors, ideations, or emotional responses, facilitating the nuanced execution of diverse cognitive tasks, inclusive of decision-making processes and goal-oriented actions (*6*, *7*). As the hippocampus serves as a hub for learning and memory, its role and the nature of its involvement in the aforementioned processes have garnered widespread attention (*8**–**12*). Current consensus postulates that neural plasticity is likely the principal neural mechanism supporting the hippocampus’s role as a center for learning and memory (*13*, *14*). This plasticity spans multiple dimensions, including dendritic, axonal, synaptic, and circuit plasticity.

Adult neurogenesis, a captivating ability inherent to the hippocampus, not only endows it with heightened neural plasticity but also offers fresh insights into its function in learning, memory, and other advanced cognitive tasks. Research has shown that persistent neurogenesis occurs in the hippocampal dentate gyrus (DG) of both animals and humans, at least extending into middle-aged individuals (*15*, *16*). During their pivotal first 1 to 1.5 months of age, these newborn neurons (NBNs) exhibit unique physiological properties, marked by heightened excitability and plasticity (*17*). This equips them with the capability to recruit inhibitory GABAergic interneurons, positioning them as suppressors of the local mature granule cell activity in the DG (*18*, *19*). In hippocampal-dependent cognitive processes, various studies have substantiated the instrumental role of these NBNs, using techniques such as tissue lesioning, x-ray ablation, and optogenetics, in the encoding of novelty, extinction of fear memories (*20*, *21*), and the handling of spatiotemporal information (*22*, *23*). As NBNs continuously integrate into existing neural circuits, their contributions to advanced cognitive functions, including reversal learning (*24*), active forgetting (*25*), and pattern separation (*26*), are consequently realized (*27*, *28*). Concurrently, the neural network restructuring triggered by the incorporation of NBNs can potentially destabilize prior memory circuits, catalyzing certain forms of forgetting (*29*, *30*), providing a structural foundation for cognitive flexibility. Nevertheless, due to the sparse distribution of NBNs and limited observation tools (*31*), targeted modulation remains elusive, curtailing not only the unveiling of their distinctive roles but also a comprehensive description of their interactions within the hippocampus and the broader brain network (*32*). As we deepen our understanding of the functional heterogeneity exhibited along the hippocampal longitudinal axis (*33*, *34*) and the physiological impacts of emotional stress on NBNs (*35*, *36*), prior interpretations of the presence and functional importance of NBNs may need revision. Moreover, the relationship between NBNs and local excitation/inhibition (E/I) balance remains intriguing. Valuable studies suggest that beyond merely their abundance, the functionality of these neurons is tightly governed by the balance between gamma-aminobutyric acid-ergic (GABAergic) and glutamatergic inputs (*15*, *37*). Perturbations in this E/I balance prove crucial in shedding light on certain clinical conditions, notably Alzheimer’s disease (*38*). Yet, comprehensive data on how NBNs delicately modulate the DG E/I dynamics are scant. Thus, a pressing challenge lies in visualizing the contributions of NBNs to the hippocampal E/I network in the most physiologically authentic conditions, offering an intuitive portrayal of their functionality, operational periods, and spheres of influence.

In this study, by combining features from two transgenic systems (*39**–**42*), we developed a transgenic mouse model, NBN-TeTX (newborn neuron–tetanus toxin expressing), that can noninvasively silence the functionality of NBNs. Through multiple reversal learning tasks (*31*, *43*), we confirmed the impairments in impulse inhibition and cognitive flexibility induced by silencing NBNs. Concurrently, task-based functional magnetic resonance imaging (tb-fMRI) visually demonstrated the impact of silencing NBNs on the activation patterns of the hippocampus during tasks (*44*, *45*). By integrating resting-state fMRI (rs-fMRI) functional connectivity analysis with investigations into the activity of parvalbumin-positive interneurons and mossy cells in the DG under various conditions (*46*, *47*), we postulate that NBNs play an integral role in modulating the E/I balance among the dorsal DG and contribute to the functional segregation between the dorsal and ventral hippocampus. These findings offer insights and a foundation for further elucidating the contributions of the hippocampus and NBNs to inhibitory control, cognitive flexibility, and spatiotemporal perception.

## RESULTS

### NBN-TeTX mouse model for noninvasive NBN silencing

**Fig. 1.**
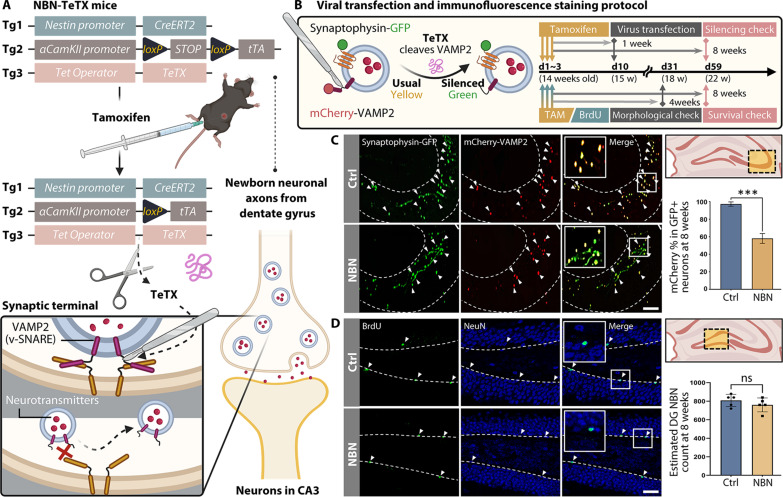
Characterization of the NBN-TeTX mouse model for noninvasive NBN silencing. (**A**) NBN-TeTX mice are transgenic, carrying three recombinant genes. TeTX inhibits NBN functionality by cleaving synaptic vesicle VAMP2 at neural terminals, thus restricting neurotransmitter release. (**B**) Fluorescent vector viral transfection method to validate the silencing rate. Synaptic vesicles at terminals are labeled with coexpressed Synaptophysin-GFP and mCherry-VAMP2. A week after tamoxifen cessation, retroviral injections are given, with perfusion and staining after 8 weeks. Typically, colocalized VAMP2 and Synaptophysin in virus-transfected terminals display an overall yellow appearance due to combined mCherry (red) and GFP (green). In terminals with TeTX, VAMP2 cleavage separates mCherry from the synaptic vesicle membrane, resulting in only green GFP fluorescence. (**C**) In the CA3 area of NBN mice, ~40% of NBN synaptic terminals showcase dislodged mCherry, in contrast to nearly none in the control group. Arrows highlight synapses cofluorescing Synaptophysin-GFP and mCherry-VAMP2 (Ctrl: mean = 97.265 ± 1.382, NBN: mean = 58.003 ± 3.366, *n* = 3 per group; ***P < 0.001, unpaired *t* test; slice thickness: 40 μm; scale bar, 100 μm). (**D**) After 8 weeks of tamoxifen administration, BrdU/NBN coimmunofluorescence staining indicates that the mature NBN count in the NBN-TeTX group is not significantly different from the Ctrl group. Arrows pinpoint hippocampal DG NBNs costained with BrdU/NeuN (Ctrl: average = 733 ± 32, NBN: mean = 690 ± 34, *n* = 5 per group, unpaired *t* test; slice thickness: 40 μm; scale bar, 50 μm). Panels (A) and (B) were created with BioRender.com.

Our primary focus is on the unique properties of adult-born neurons (3 to 6 weeks old) in memory, learning, and cognition. Thus, we have developed a tri-transgenic mouse model named NBN-TeTX (NBN; Fig. 1A and table S1). In this model, administering tamoxifen to adult mice specifically induces the activation of *CreERT2* only in neural stem/progenitor cells (NSCs/NPCs) that express *Nestin*. Moreover, as the *Cre*-expressing stem cells mature to approximately 2.5 to 3 weeks of age, the onset of α*CamKII*-mediated *tTA* expression drives the production of TeTX, ensuring that TeTX does not affect NSCs at early developmental stages. TeTX inhibits the fusion of neurotransmitter vesicles with the presynaptic membrane by cleaving the synaptic vesicle protein VAMP2, ultimately suppressing the neurotransmitter release associated with the activity of NBNs during learning, memory, and cognitive tasks.

We first confirmed the expression of TeTX in synaptic terminals of NBNs through virus-mediated immunofluorescence staining (Fig. 1B) and verified that approximately 40% of NBNs in the NBN mouse model were functionally silenced (Fig. 1C). Specifically, after administering tamoxifen to 14-week-old adult mice and waiting 1 week for TeTX to be fully expressed and accumulate in the synaptic terminals of labeled NBNs, we then proceeded with retroviral injections to respectively label Synaptophysin [green fluorescent protein (GFP)] and VAMP2 (mCherry). Seven weeks after viral injection (i.e., when NBNs are around 8 weeks old and fully matured), we conducted tissue slicing and immunofluorescence staining. Results indicated that, in the CA3—the primary projection area of the DG NBNs—about 40% of the synaptic terminals in NBN mice showed residual green fluorescence of Synaptophysin-GFP due to the shedding of mCherry-VAMP2, compared to the control group. In contrast, synaptic terminals in the control group were almost entirely of a normal yellow color.

Simultaneously, to ascertain whether the NBN mouse model induces functional inhibition of NBNs without affecting their survival and/or overall neurogenesis levels, we performed 5-bromo-2′-deoxyuridine (BrdU)/NeuN immunofluorescent costaining in the subgranular zone of the DG 8 weeks after simultaneous administration of tamoxifen and BrdU. The results indicated no significant difference in the number of surviving mature NBNs between the NBN group and the control group (Fig. 1D). Moreover, to characterize neurogenesis during the pivotal 3- to 6-week maturation window, we quantified NSCs (GFAP^+^/Sox2^+^), NPCs (GFAP^−^/Sox2^+^), immature NBNs (DCX^+^/NeuN^−^), and mature NBNs (BrdU^+^/NeuN^+^) 4 weeks after tamoxifen administration, finding no significant variations in these populations (fig. S1, A and B). When further analyzing the morphology of immature neurons at this stage, we observed a significant reduction in the average number of dendritic branches in the NBN group, as well as slight decreases in average dendritic length and total dendritic length, while no significant differences were found in the average primary dendrite length or the proportion of dendrites across different length intervals (fig. S1C).

In summary, we have validated that the NBN-TeTX mouse model provides a noninvasive functional silencing window for NBNs from their birth to maturation while avoiding macroscopic or significant alterations to the hippocampal architecture. By modulating the time of tamoxifen administration, this model enables temporally specific functional silencing of NBNs without affecting NSCs during early developmental stages.

### Spatial reversal learning impairment by NBN silencing

**Fig. 2.**
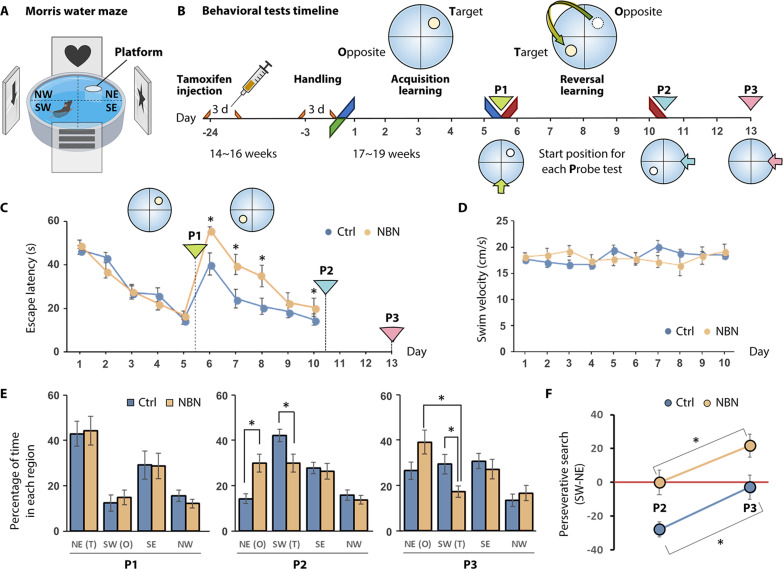
Impaired spatial reversal learning during Morris water maze. (**A**) Morris water maze (MWM) experimental setup schematic, created with BioRender.com. (**B**) MWM experiment protocol. Probe tests P1 and P2 occurred immediately after the learning periods on days D5 and D10, respectively. P3 was scheduled for day 13. (**C**) Escape latency progression (in seconds) over the 10-day training. Significant differences between the groups were observed only during the reversal learning phase (D6 to D10: *P < 0.05, two-way ANOVA; mean ± SEM). (**D**) No significant difference in movement speed (cm/s) was found throughout the 10-day training (two-way ANOVA). (**E**) Percentage of time both mouse groups spent exploring each quadrant during the spatial probe trial: At P1, exploration time in each quadrant showed no significant difference between the groups (NE: *P* = 0.8808, two-way ANOVA). At P2, differences were seen in time spent in the NE and SW quadrants (NE: *P* = 0.0045, SW: *P* = 0.0285; *P < 0.05, two-way ANOVA). At P3, only the time in the SW quadrant differed between groups (SW: *P* = 0.0232, two-way ANOVA). Within the NBN group, significant differences arose between time spent in the NE and SW quadrants (Ctrl: *P* = 0.6394, NBN: *P* = 0.0015; *P < 0.05, two-way ANOVA). (**F**) Changing preference of both groups for the NE and SW quadrants, where the platform was located, from P2 to P3 (Ctrl: *P* = 0.0099, NBN: *P* = 0.0465; *P < 0.05, two-way ANOVA).

We used a reversal learning task in the Morris water maze to ascertain the impact of silencing NBNs on cognitive flexibility (Fig. 2A). Thirteen male NBN-TeTX mice (NBN) and 11 male control mice (Ctrl) received tamoxifen injections at 14 weeks of age and, 3 weeks later, underwent 5 days of acquisition learning (D1 to D5) followed by 5 days of reversal learning (D6 to D10). Spatial probe tests were scheduled accordingly on day 5 (after acquisition learning), day 10 (after reversal learning), and day 13 (Fig. 2B; see also fig. S2A). Throughout the 10-day training period, there was no significant difference between the two groups in swimming speed (Fig. 2D). As shown in Fig. 2C (D1 to D5), there was no significant difference between the NBN and Ctrl groups in escape latency during the first 5 days of acquisition learning. In the first spatial probe test (P1), both groups showed no significant difference in the time spent in the target quadrant (NE), indicating that silencing 3- to 6-week-old integrating and maturing NBNs did not exhibit a significant difference in initial spatial learning (Fig. 2E, P1).

During reversal learning (D6 to D10), the platform was moved to the SW quadrant. Throughout the 5-day reversal learning process, the NBN group consistently displayed a significantly longer escape latency compared to the Ctrl group, although this gap gradually diminished (Fig. 2C, D6 to D10). In the second spatial probe test (P2), the two groups of mice displayed differential area preferences. Ctrl mice explored the new location of the platform (SW) more, while the NBN group still favored the original platform location (NE) (Fig. 2E, P2). The third spatial probe test (P3) was conducted on day 13 to assess the final long-term memories formed by both groups after 10 days of initial and reversal learning. At the P3 time point, Ctrl mice spent an equal amount of time exploring the previous two target locations (NE and SW), while the NBN group still displayed a pronounced preference for the NE quadrant (Fig. 2E, P3). By comparing the percentage search time for the NE and SW quadrants in P2 and P3 between the two groups, we observed a shift in preference in both groups. The Ctrl group transitioned from a preference for the platform’s initial NE quadrant to an equal preference for both quadrants where the platform had been located, while the NBN group maintained its attachment to the platform’s initial quadrant (Fig. 2F). These data indicate that mice exhibit an attachment to initial memories after silencing immature NBNs.

### Impulse inhibition impairment in reversal learning by NBN silencing

**Fig. 3.**
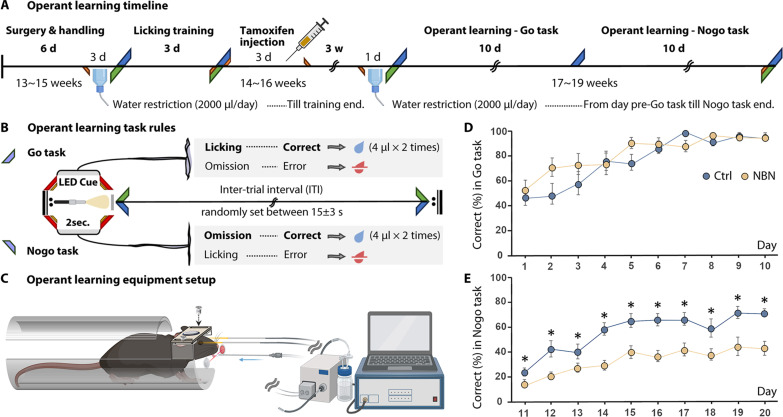
Impacted impulse inhibition in the Nogo task of operant learning. (**A**) Operant learning protocol based on light cues: 11 NBN and 9 Ctrl mice participated. A 3-day light reflex training preceded tamoxifen administration to ensure consistent light cue water reward conditioning across both groups. (**B**) Rule setup for the Go and Nogo tasks. (**C**) Schematic of the operant learning apparatus setup, created with BioRender.com. Mice are secured on a custom operant learning bed via a pre-attached head plate. (**D**) Each group’s accuracy trend across the 10-day Go task. By day 10, both groups consistently achieved over 95% accuracy, showing no marked intergroup differences (day 9 accuracy: Ctrl: 96.37%, NBN: 94.96%; day 10: Ctrl: 95.72%, NBN: 95.68%; two-way ANOVA; Ctrl: *n* = 9, NBN: *n* = 11; mean ± SEM). (**E**) Accuracy trends for each group during the 10-day Nogo task. The NBN group’s accuracy consistently trailed the Ctrl group throughout (genotype: *F*_1__,__18_ = 16.6545, *P* = 0.0007; *P < 0.05, two-way ANOVA; Ctrl: *n* = 9, NBN: *n* = 11; mean ± SEM).

To further confirm the effect of silencing NBNs on mice’s cognitive flexibility, we conducted an operant learning experiment using the Go/Nogo paradigm (Fig. 3, A and B). This was done to evaluate the mice’s inhibitory control by varying the conditions of reinforcement learning. Initially, we performed a head plate fixation surgery (Fig. 3C) on 11 male NBN mice and 9 male Ctrl mice aged 13 to 15 weeks and conducted a 3-day handling, initiating water restriction on the last day of the handling. This was followed by 3 days of light-lick training. The light cue was always on, and it briefly turned off when the mouse licked, immediately followed by a water reward. This established a connection between the light stimulus (Cue) and the water reward (Reward) before silencing the function of NBNs. No significant difference in the total number of licks was observed in the 5-min post-training test (fig. S3A). Tamoxifen injections were then administered to mice aged 14 to 16 weeks, and 3 weeks later, a 10-day Go task and a 10-day Nogo task began.

During the D1 to D10 Go task (Fig. 3B, Go task), both the NBN and Ctrl groups of mice gradually improved their accuracy rate (Correct%) of correct licking over the 10 days, reaching around 95% by the end of the task (Fig. 3D). During this period, there was no significant difference in the increasing trend of accuracy between the two groups. The daily total number of licks (fig. S3B), the number of incorrect licks before reaching 100 correct licks daily (fig. S3C), and the daily response latency (reaction speed to the light stimulus) (fig. S3D) also showed no significant differences between the groups.

Subsequently, during the D11 to D20 Nogo task (Fig. 3B, Nogo task), the mice were required to inhibit their learned impulse to lick water during the light stimulus and wait for the light cue to end to receive the water reward. We observed that throughout the 10-day Nogo task, the NBN group displayed a behavioral inhibition defect in suppressing licking. Despite not receiving a water reward, they frequently licked water during the light cue, causing their accuracy rate to consistently lag that of the Ctrl group (Fig. 3E). This suggests that, in reinforcement learning tasks with changing conditions, silencing immature NBNs appears not to affect the learning of initial rules. However, it significantly affects impulse inhibition, making new reinforcement learning challenging or requiring more extended periods to achieve.

### Hippocampal activation shifts during initial and reversal learning via fMRI

To further understand the functional impact of the silencing of NBNs on the hippocampal activation state, we first combined the operant learning experiment with fMRI. By using task-based fMRI (tb-fMRI), we analyzed the activity state of the hippocampus of awake mice in real time and noninvasively during the Go/Nogo tasks.

**Fig. 4.**
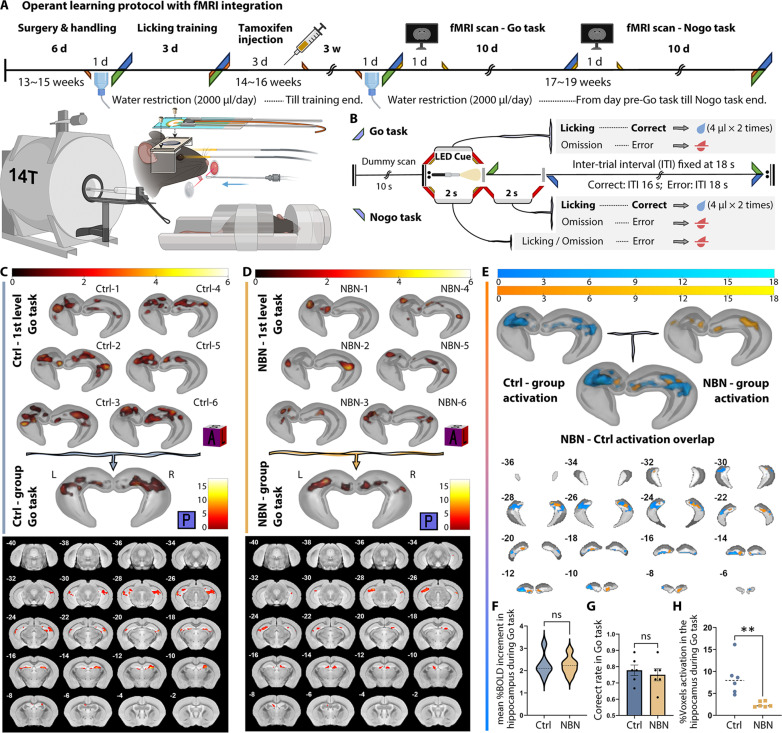
Hippocampal activation in Go task via tb-fMRI. (**A**) Operant learning protocol with light cues inside fMRI captures 18 trials over 6 min on day 1 of Go and Nogo tasks. The schematic for this protocol was created with BioRender.com. (**B**) Go and Nogo task rules for fMRI: 20-s trials. In the Nogo task, post-light cue licking grants a reward, countering freezing due to fMRI conditions. Licking within 2 s after the light cue is considered correct and rewarded in the Nogo task. Additionally, brain activity during this period is regarded as the active state corresponding to the baseline in the analysis. A hippocampal anatomical mask was utilized to accurately track activation responses. (**C**) 3D rendering of hippocampal activity in the Ctrl group’s individual and intra-group Go task (pFDR < 0.05); posterior coronal visualization details section’s bregma relation. Second level: peak *T* value: 15.25, cluster extent (KE) = 448. (**D**) 3D rendering of hippocampal activity in the NBN group’s individual and intra-group Go task (pFDR < 0.05); posterior coronal visualization details section’s bregma relation. Second level: peak *T* value: 14.29, cluster KE = 216. (**E**) Overlaid second-level intra-group results for Ctrl and NBN during Go task highlight spatial activation differences. (**F**) Both groups exhibit elevated %BOLD in hippocampus 2 to 4 s after the light cue. No significant difference observed (Ctrl: 2.258, NBN: 2.236; *n* = 6 per group; two-tailed, unpaired *t* test). (**G**) Correct licking rate in fMRI-captured Go task trials: Ctrl: 0.7778, NBN: 0.7500; no significant group difference (*n* = 6 per group; two-tailed, unpaired *t* test, mean ± SEM). (**H**) Ratio of activated to total hippocampal voxels per individual calculated. Ctrl’s Go task activated voxel percentage significantly higher than NBN’s (Ctrl: 8.524%, NBN: 2.456%; **P < 0.005, two-tailed, unpaired *t* test; *n* = 6 per group).

Six male mice each from NBN and Ctrl groups (aged 13 to 15 weeks) underwent head plate fixation surgery. Apart from being trained in the fMRI device with a 90-dB spin-echo echo-planar imaging (SE-EPI) scanning sound to acclimate them to the environment, all other procedures were as described above (Fig. 4A). On the first day of the Go task and the first day of the Nogo task, we scanned the two groups of mice with fMRI (6 min), with specific task rules as shown in Fig. 4B. On the basis of hippocampal anatomical structures, we set up a hippocampal mask and conducted a region of interest (ROI) analysis. We assessed hippocampal activity changes after the light cue (2 to 4 s) against baseline (average of the last 4 s per trial).

In the Go task, the licking accuracy rate of both groups during the 18 trials in 6 min has no significant difference (Fig. 4G); however, the fMRI analysis results were intriguing. From the first-level analysis (individual) results of the hippocampal activation pattern of each individual in both groups, we observed similar activation patterns within the groups (Fig. 4, C and D, top). By calculating the percentage of significantly activated hippocampal voxels, we confirmed that the activation range of the Ctrl group’s hippocampus was significantly larger than that of the NBN group (Fig. 4H). Through the three-dimensional (3D) rendering of the second-level intra-group analysis results (Fig. 4, C and D, bottom; see also fig. S4B, left) and the overlaid display of the activated voxel coronal montage (Fig. 4E), one can intuitively observe the spatial distribution differences in hippocampal activation. The second-level intergroup analysis did not return voxels with significant differences. We further extracted the blood oxygen level–dependent (BOLD) signal values throughout the scan and calculated their percentage change (%BOLD change). The results suggested that there was no significant difference in the BOLD intensity elevation level during the Go task between the two groups. These results indicate that silencing immature NBNs during the Go task did not cause significant differences in hippocampal activity levels and behavioral performance but did result in a sharp reduction in spatial distribution.

**Fig. 5.**
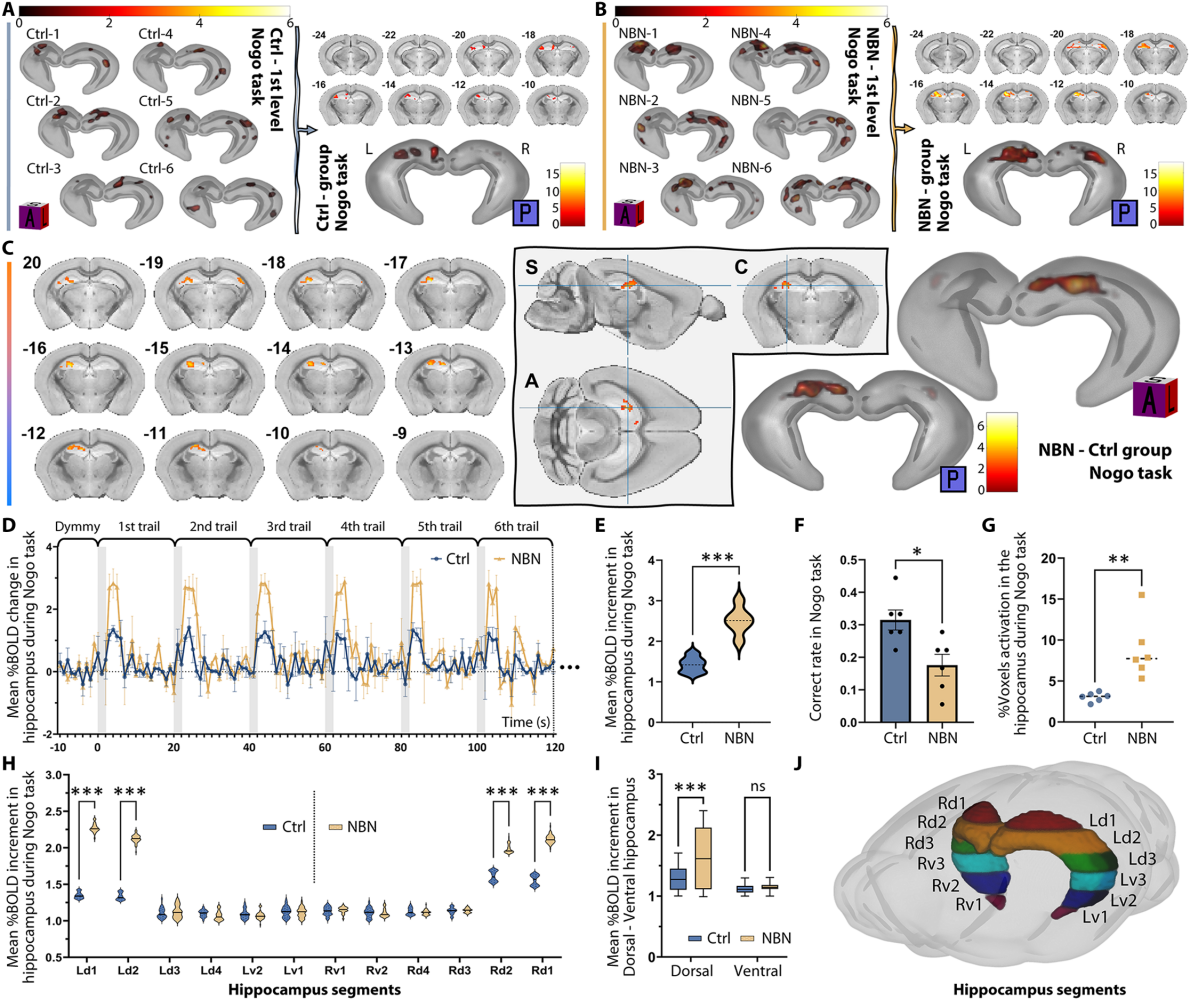
Hippocampal activation in Nogo task via tb-fMRI. (**A**) 3D rendering of hippocampal activity in the Ctrl group’s individual and intra-group Nogo task (group: peak *T* value: 6.11, pFDR < 0.05). (**B**) 3D rendering of hippocampal activity in the NBN group’s individual and intra-group Nogo task (group: peak *T* value: 18.41, pFDR < 0.05). (**C**) Second-level intergroup analysis results for the Nogo task (NBN > Ctrl, peak *T* value: 5.28, pFDR < 0.05). (**D**) Hippocampal %BOLD signal changes are plotted over the initial 2-min frame during Nogo task (mean ± SD, *n* = 6 per group). (**E**) NBN group exhibit significantly elevated %BOLD in hippocampus against the Ctrl group within 2 to 4 s after the light cue (***P < 0.001, two-tailed, unpaired *t* test). (**F**) In the Nogo task, the NBN group’s accuracy rate in the initial 18 trials was significantly lower than that in the Ctrl group (*P < 0.05, two-tailed, unpaired *t* test, mean ± SEM). (**G**) Ctrl’s Nogo task activated voxel percentage became significantly lower than that of the NBN group (Ctrl: 3.045%, NBN: 8.776%; **P < 0.005, two-tailed, unpaired *t* test; *n* = 6 per group). (**H**) BOLD signal measurements from various layers of the hippocampus reveal notable intensity changes, especially in the NBN group’s d1 and d2 layers (***P < 0.001, two-way ANOVA). (**I**) Evaluations of the average BOLD intensity changes between the dorsal and ventral hippocampus indicate predominant dorsal activation in the NBN group during the Nogo task (dorsal: ***P < 0.001, two-way ANOVA). (**J**) An illustration demarcates the mask used for BOLD signal extraction. The hippocampus is segmented into layers, with a breakdown of the dorsal (d1 to d4) and ventral (v1 to v2) regions. Both sides of the hippocampus are assessed independently.

However, on the first day when the rule switched to the Nogo task, the activation patterns of the two groups underwent a marked reversal. First, similar to the results of the Nogo task in the operant learning experiment conducted outside of the fMRI, during the initial 6 min covered by the fMRI scan, which consisted of 18 trials, although neither group could achieve a high accuracy rate, the licking accuracy rate of the Ctrl group was still higher than that of the NBN group (Fig. 5F). Subsequently, in the individual analysis, we observed dissimilarity in the spatial distribution of hippocampal activation between the two groups (Fig. 5, A and B, left). The activation range of the Ctrl group’s individuals during the Nogo task was significantly smaller than that of the NBN group (Fig. 5G) and was smaller than their own range during the Go task (fig. S4B). The NBN group, on the other hand, displayed a larger hippocampal activation range than its own performance during the Go task. Further, the intra-group analysis highlighted the dissimilarity between the two groups’ hippocampal activities (Fig. 5, A and B, right; see also fig. S4B, right). Subsequently, in the intergroup analysis, we confirmed a significant difference in hippocampal activity between the NBN and Ctrl groups (Fig. 5C). Notably, whether looking at the spatial distribution of intragroup results or the spatial distribution of intergroup differences, it seems to be mainly located in the dorsal region of the hippocampus.

To speculate on the reason for the activity differences concentrated in the dorsal hippocampus, we first calculated the percentage change in BOLD intensity of the two groups during the Nogo task. The results showed that within 2 s after the end of the light cue, during the peak period of the BOLD signal, the NBN group was significantly higher than the Ctrl group (Fig. 5, D and E). Then, we divided the hippocampus along the longitudinal axis into six layers (Fig. 5J). The upper two-thirds (four layers) were considered the dorsal hippocampus (d1 to d4), and the lower one-third (two layers) was considered the ventral hippocampus (v1 to v2). We then extracted the BOLD signals from each layer of the bilateral hippocampus and calculated the percentage change. The results showed that during the Nogo task, the dorsal hippocampal layers d1 to d2 of both groups contributed mainly to the rise in the BOLD signal, but the NBN group exhibited a significantly higher activation relative to Ctrl. However, no significant differences were observed in the entire ventral hippocampus between the two groups (Fig. 5, H and I).

In summary, tb-fMRI successfully visualized the transition in hippocampal activity patterns during the Go/Nogo task. On the first day of the Go task, prominent activity observed in both mouse groups indicated hippocampal engagement in detecting novelties and initiating learning. However, NBN silencing led to a significant reduction in the spatial distribution of activated regions. Although no behavioral or mean %BOLD intensity change differences were observed in the NBN group during the Go task, the reduced activation areas in conjunction with poorer subsequent Nogo task performance indicated changes in initial learning strategies and intensities due to impaired NBN function. In the Nogo task, the Ctrl group completed the new rule learning with considerably less hippocampal activity compared to the heightened neural activity seen in the NBN group. Notably, the primary differences were observed in the dorsal hippocampus.

### Enhanced dorsal and ventral DG functional connectivity with NBN silencing

To understand the heightened dorsal activation in the NBN group during the Nogo task and the effect of NBN silencing on the hippocampal functional network, we conducted a 6-min rs-fMRI scan on 14 male NBN mice and 14 male Ctrl mice each, 3 weeks after tamoxifen injection while they were under anesthesia.

**Fig. 6.**
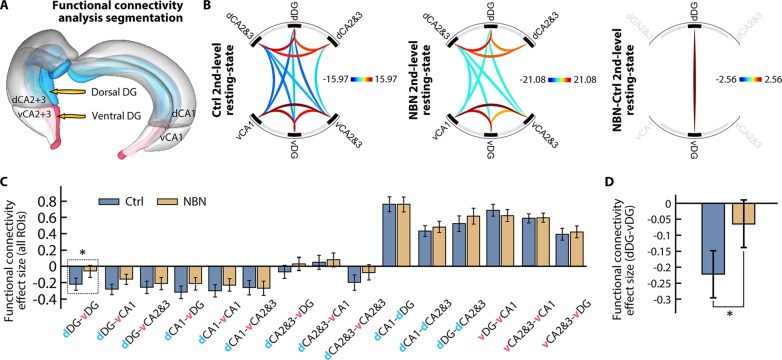
Functional connectivity with NBN silencing via rs-fMRI. (**A**) Schematic of ROI settings for the rs-fMRI functional connectivity analysis. The bilateral hippocampus is segmented based on anatomy and dorsal-ventral distinctions: dDG (upper two-thirds), vDG (lower one-third), dCA1, vCA1, dCA2&3, and vCA2&3. (**B**) From left to right: Second-level intra-group functional connectivity for the Ctrl and NBN groups, second-level intergroup analysis, and a chord diagram for NBN > Ctrl (*T*_26_ = 2.56, FDR-corrected *P* < 0.05 ROI-level threshold (ROI mass/intensity), *n* = 14 per group). (**C** and **D**) Comparison of *z* values (Fisher-transformed) between the groups. A notable difference in functional connectivity between NBN and Ctrl was seen in dDG-vDG (β = 0.16, *T*_26_ = 2.56, *P = 0.016, two-sample *t* test, mean ± SEM; *n* = 14 per group).

In ROI-ROI functional connectivity analysis, we divided the hippocampus into DG, CA1, and CA2&3, defining six volumes of interest (VOIs) based on dorsal (d) and ventral (v) sections (Fig. 6A). In the second-level intra-group analysis, we observed differences in the hippocampal functional connectivity patterns between the NBN and Ctrl groups. Notably, in the Ctrl group, there was significant negative connectivity between dDG-vDG and dCA2&3-vCA2&3, which was absent in the NBN group. The functional connectivity difference between dDG-vDG persisted in the subsequent second-level intergroup analysis (Fig. 6B). To quantify this disparity, we used Fisher’s *z* transformation to convert Pearson correlation coefficients into *z* values. As shown in Fig. 6C, for ROIs within either the dorsal or ventral areas, functional connections were predominantly positive with no significant difference between the two groups. However, for cross-region connections between dorsal and ventral, both groups displayed negative functional connectivity, but the NBN group exhibited a general trend of reduced negative values. Specifically, there was a significant difference between the NBN and Ctrl groups in dDG-vDG correlations (Fig. 6D).

Our findings suggest that silencing the NBNs altered the functional connectivity patterns between the dorsal and ventral regions of the hippocampus. Typically, negative functional connectivity implies that as one region becomes more active, the other becomes less active. However, in the NBN group, this negative connectivity between the dorsal and ventral DG was notably reduced, leading to an increase in synchronous activity between these regions. Such synchronicity indicates stronger or more enhanced functional connectivity, which, in this context, seems to be an abnormal consequence of NBN silencing.

### DG E/I balance alterations by NBN silencing

**Fig. 7.**
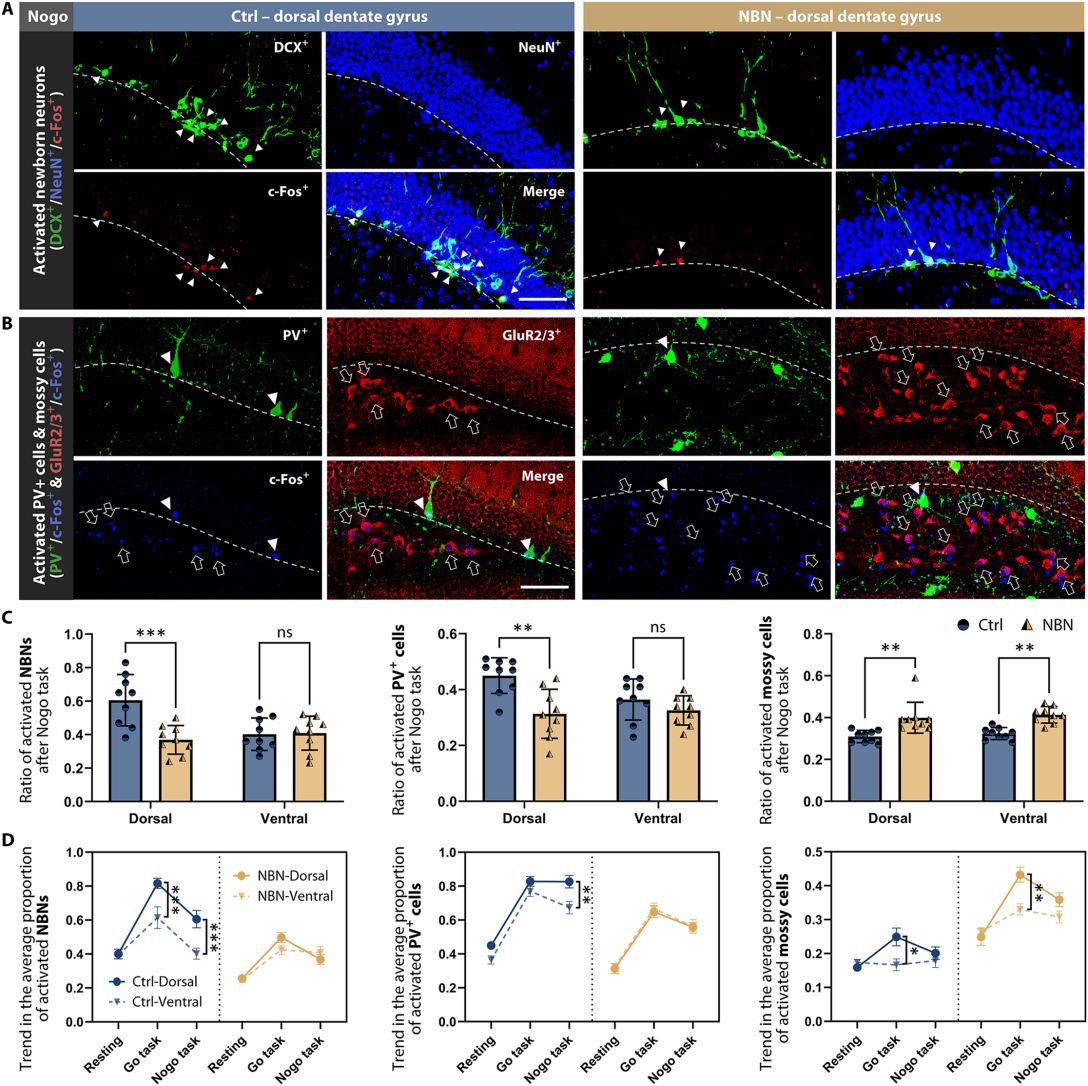
Dynamic changes in DG E/I components activity. (**A**) Immunofluorescence of NBN activity (DCX^+^/NeuN^+^/c-Fos^+^) after Nogo task. The ratio of c-Fos cells to the overall count of relevant cells in the DG of each slice was determined (*n* = 3 per group; scale bar, 100 μm). (**B**) Immunofluorescence of parvalbumin-positive GABAergic interneuron (PV^+^/c-Fos^+^) and mossy cell (GluR2/3^+^/c-Fos^+^) activity after Nogo task (*n* = 3 per group; scale bar, 100 μm). (**C**) c-Fos^+^ NBNs, PV^+^ interneurons, and mossy cell proportions in the hippocampal DG after Nogo task for both groups (*P < 0.05, **P < 0.01, ***P < 0.001, two-way ANOVA, mean ± SEM). (**D**) Dorsal-ventral changes and trends in activated NBNs, PV^+^ interneurons, and mossy cells in the hippocampal DG under each condition (NBN: *n* = 3; *P < 0.05, **P < 0.01, ***P < 0.001, two-way ANOVA, mean ± SEM; *n* = 3 per group at each condition).

Hyperactivation of the dorsal hippocampus in the NBN group during reversal learning hints at changes in the E/I balance. To explore this, we examined c-Fos expression in various cell types, including NBNs, inhibitory component parvalbumin-positive GABAergic interneurons, and excitatory component mossy cells, in the hippocampus’s DG after the Nogo task (Fig. 7, A and B). We also assessed this under post-Go task and resting-state conditions.

After the Nogo task, the NBN group displayed a significant drop in c-Fos^+^ NBNs (DCX^+^/NeuN^+^) in the dorsal DG compared to the Ctrl group. However, this difference was not present ventrally (Fig. 7, A and C; see also fig. S5, A and B). Additionally, in the NBN group, the number of c-Fos^+^ PV^+^ (parvalbumin^+^) interneurons in the dorsal region was reduced compared to the Ctrl group. Meanwhile, the expression of c-Fos^+^ in mossy cells surpassed that of the Ctrl group in both dorsal and ventral areas (Fig. 7, B and C; see also fig. S5, A and B).

After the Go task, the NBN group had fewer c-Fos^+^ NBNs in both regions compared to the Ctrl group. In the resting state, NBN activity in the NBN group remained consistently lower across both regions than in the Ctrl group. c-Fos^+^ PV^+^ interneuron activity in the NBN group showed a decline dorsally in the resting state and after the Go task. Conversely, c-Fos^+^ mossy cell increased across both domains.

As experiments progressed from resting to the Nogo task, NBN activity during tasks increased relative to the resting phase for both groups. Yet, this increase was always less pronounced in the NBN group (Fig. 7D). After the Nogo task in the Ctrl group, dorsal PV^+^ interneuron activity was higher than in the ventral area, but this was not the case in the NBN group. Mossy cell activity in the NBN group remained consistently elevated across both dorsal and ventral hippocampus.

## DISCUSSION

The unique contribution of NBNs to cognitive function and behavioral regulation is far more impressive than their sparse numbers would suggest. Adult hippocampal neurogenesis, while widespread in mammals, including humans, remains controversial mainly due to the restricted techniques for modulation and observation in humans (*48*, *49*). Previous research highlighted the role of NBNs in novelty encoding, spatial navigation, and learning, primarily by observing natural aging, manipulating their numbers or using x-ray irradiation to disrupt them (*27*, *50**–**54*). However, inconsistencies in these findings (*55*, *56*) underscored the need for a noninvasive method to modulate NBN numbers without altering hippocampal anatomy. Recognizing these challenges, our study used the engineered NBN-TeTX tri-transgenic mouse model to provide clarity. By integrating the *Nes-CreERT2* and *TetO-TeTX* systems, we achieved targeted and temporal modulation of NBNs. The *Nes-CreERT2* system enables precise temporal control, allowing for permanent labeling of NSCs/NPCs at specified time points by modulating the timing of tamoxifen administration (*39*). Subsequently, in the *TetO-TeTX* system, the tetanus toxin is only activated as these labeled NSCs/NPCs gradually mature into neurons and initiate the expression of α*CamKII*. Previous work has confirmed that α*CamKII* expression in adult-born granule cells begins 2.5 to 3 weeks after differentiation, aligning with their synaptic integration into hippocampal networks (*57*). TeTX then specifically suppresses activity-dependent neurotransmitter release by cleaving VAMP2, effectively inhibiting synaptic transmission and silencing the function of NBNs (*41*). Correspondingly, our behavioral experiments in the 3- to 6-week window after tamoxifen are designed to target this critical phase of synaptogenesis and functional maturation, examining the effects of NBN silencing on the hippocampus.

Furthermore, previous study on the temporal expression pattern of doublecortin (DCX) in neurogenic regions of the adult rat brain elucidates that DCX not only is present during proliferation and migration but also accompanies dendritic elongation and branching of maturing NBNs. This transient expression pattern of DCX during the morphological development and functional maturation of NBNs emphasizes its effectiveness as a marker for detecting adult neurogenesis (*58*). Our morphological analysis of immature NBNs marked with DCX^+^/NeuN^−^ revealed dynamic changes in dendritic morphology in the NBN group after functional silence. Although it is not yet possible to locate this change in the specific NBN individuals affected by the silence or the overall change in NBN, the reduction in the number and length of dendritic branches still implies its adverse effect on normal hippocampal internal network connections and complexity. It is also noteworthy that the NBN-TeTX mouse model enables us to target and modulate NBNs without affecting early-stage NSCs, whose impairment can hinder neurogenesis and disrupt adult neural networks. Moreover, increasing evidence suggests that NSCs regulate their microenvironment through the secretion of cytokines and proteins—their “secretome”—which modulates immune responses and tissue repair and supports surrounding cell functions (*59*). Direct alterations to NSCs might adversely affect these critical functions and exert long-term effects on the entire neural environment, thereby skewing the true functions of NBNs. By preserving NSC integrity, the NBN-TeTX model allows the assessment of functions solely dependent on adult-born neurons.

The DG, as the hub of neurogenesis, plays a pivotal role in hippocampal-dependent tasks, notably pattern separation (*60**–**62*). A previous study that used a strategy conceptually opposite to ours and similarly focused on the behavioral and cognitive functions of NBNs found that enhancing the survival rate of NBNs enhanced pattern separation ability and increased exploratory behavior (*63*). Although the strategies diverge, our work similarly illustrates the pivotal role of NBNs in impulse control and cognitive flexibility and contributes to the growing body of evidence that underscores the role of NBNs in differentiating highly similar situations (*26*, *64*). Pattern separation inherently requires the discrete encoding of similar data, necessitating sparse activity patterns within the DG (*65*). NBNs, compared to their mature dentate granule cell counterparts, may provide transient support for this sparse activity during specific phases (*66*). We propose that NBNs modulate hippocampal activation patterns, potentially influencing the DG’s E/I balance.

Moreover, our research adds nuance to the ongoing debate surrounding the distinct functionalities of the dorsal and ventral parts of the hippocampus, particularly in relation to cognitive flexibility (*34*, *67**,* *68*). Both our team and others are in the process of establishing experimental systems to gather fMRI data from mice engaged in the Go/Nogo tasks (*43*, *44*). Our tb-fMRI results show a marked decrease in the range of hippocampal activation during initial learning, a consequence of impaired NBN function. This reduction likely stems from inadequate activation of downstream neurons and neural circuits, given the crucial role of NBNs in novelty detection. These findings echo our previous morphological observations, where reduced dendritic branching in NBNs highlighted an indirect connection between structural changes and functional outcomes in hippocampal neurogenesis. Additionally, the absence of significant differences in hippocampal activation intensity does not rule out transient compensatory mechanisms, which might temporarily maintain intensity levels despite a reduced activation range. Nonetheless, this reduction in activation range could still impair memory consolidation and retrieval, adversely affecting behavioral performance. Despite potential compensatory efforts, the diminished range of activation is a notable concern for hippocampus-dependent cognitive functions. In response, particularly during reversal learning, the hippocampus may increase its efforts, leading to a more extensive or intensified activation pattern, as it adapts and restructures new memories. However, the fact that hyperactivation is predominantly distributed in the dorsal hippocampus and not significantly present in the ventral region raises some interesting issues. Such shifts seem to not only alter initial learning strategies and intensities but also disrupt the typical connectivity within the hippocampus, which could potentially be the essence of compromised cognitive flexibility during reversal learning. We draw attention to a compelling study that underscores the effects of chronic neurogenesis suppression, which can trigger compensatory yet maladaptive reorganization within the hippocampal connectivity, especially in the cholinergic pathways (*69*). Such remodeling entails the recruitment of ventral projecting axons for dorsal neural innervation, a phenomenon that resonates subtly with our observations. Such insights bolster the argument that NBNs may be crucial in preserving the structural integrity and functional segregation of the hippocampal dorsal-ventral axis.

**Fig. 8.**
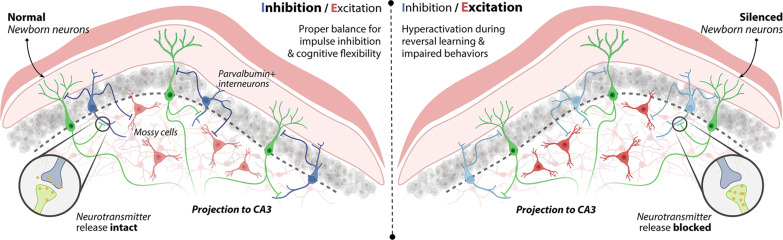
E/I imbalance in the DG following NBN silencing. With functional NBNs, the robust inhibitory input from dorsal DG PV^+^ cells ensures moderate hippocampal activation during cognitive flexibility tasks. When NBN function is compromised, reduced PV^+^ cell activity results in hippocampal hyperactivation, accompanied by increased mossy cell activity. The schematic was created with BioRender.com.

To further reveal the abnormal activation of the dorsal hippocampus in reversal learning observed by fMRI, we specifically focused on the E/I balance in the DG. Earlier research has shown that NBNs form tight connections with adjacent GABAergic interneurons and glutamatergic mossy cells (*46**,* *70**,* *71*). Among them, PV^+^ interneurons have dense dendritic structures and form synapses extensively with excitatory cells across various layers. By receiving ample excitatory impulses and monitoring excitation levels, these PV^+^ cells distribute potent inhibitory signals uniformly to surrounding neurons, playing a crucial role in maintaining the E/I balance within neural circuits (*47**,* *72**–**74*). Meanwhile, mossy cells in the hilus, serving as the first to project glutamate inputs to NBNs (*75*), are considered sentinels of the DG network (*76*). Their axons, spanning approximately 60% of the DG longitudinal axis through the commissural pathway, allow for broad projection, which also allows them to regulate the E/I balance within the DG’s granule cells (*77**–**79*). Moreover, this regulation arises not only from providing direct excitatory glutamate signals to granule cells but also from projecting to a series of GABAergic interneurons, indirectly supplying inhibitory signals to granule cells. Optogenetic and electrophysiological studies consistently indicate that NBNs synapse with hilus glutamatergic mossy cells and GABAergic interneurons, imposing feedback inhibition on the DG (*46**,* *47**,* *53**,* *63**,* *80*). Specifically, enhanced NBN survival correlates with increased excitatory drive to these interneurons (*81*), while reduced hippocampal neurogenesis diminishes feedback inhibition of DG granule cells (*82*). Our data concur with prior findings, further highlighting the central role of NBNs in neural circuit equilibrium. Notably, from as early as 3 to 4 weeks of age, NBNs seem to modulate adjacent PV^+^ cell populations, contributing to the balance between inhibitory and excitatory dynamics in the DG during reversal learning. This process involves reducing GABAergic interneuron recruitment and attenuating PV^+^ activity, which in turn amplifies mossy cell excitatory responses. These alterations in DG dynamics can subsequently affect cognitive flexibility, as illustrated in Fig. 8.

Prior research has demonstrated that animals with hippocampal damage struggle to adapt their learned behaviors in changing environments, becoming excessively influenced by past experiences (*9*). Our current work expands on this association between the hippocampus and inhibitory control, proposing a pivotal role for hippocampal NBNs. Specifically, mice with silenced hippocampal NBNs were profoundly influenced by external stimuli and entrenched behaviors, leading to a marked deficit in impulsivity control. Concurrently, despite the absence of significant behavioral differences during the initial learning phase, Go-task fMRI detected variations in hippocampal activation following NBN silencing, hinting at the underlying influence of impaired NBNs on working memory and its close tie with inhibitory control. While achieving cognitive flexibility demands a longer developmental trajectory and greater cognitive investment compared to simple memory or inhibition, it further necessitates sustained effort and training. Although the characterization of critical network nodes vital for cognitive flexibility is ongoing, the hippocampus and the prefrontal cortex (PFC), along with their bidirectional communication, are progressively viewed as central, particularly in reversal learning tasks (*27**,* *83**–**86*). Further studies on NBN silencing’s effects on hippocampal connectivity—encompassing strength, sequence, and patterns—with other cognitive control nodes will enhance our understanding of the brain’s organization of memory, forgetting, and spatial-temporal perception.

Nonetheless, certain limitations in this study warrant acknowledgment. First, given that the NBN-TeTX mouse model is recently developed, we mainly used male mice to reduce variability in behavioral and fMRI scanning/analysis stemming from gender differences, potentially not capturing female-specific responses, and underscoring the need for further exploration of sex differences. Next, the Nestin-CreER(T2) line we used may induce nonspecific recombination beyond the subgranular zone (SGZ) (*87*), suggesting possible influences on areas like the cortex, striatum, and thalamus. Considering the primary location of neurogenesis and maturation, as well as the highly hippocampus-dependent task design, we hypothesize that our results mainly stem from the functional silencing of NBNs in the SGZ. Besides, it is important to note that while our immunofluorescent staining and cellular morphological analyses provide preliminary insights into the noninvasive silencing of NBNs at a macroscopic level by our model, the prolonged blockade of NBN neurotransmission by TeTX might lead to a decline in dendritic spine density (*88*). This could potentially reduce the competitive insertion capability of NBNs in forming synapses previously established, thereby altering hippocampal structures at a microscopic level. Moreover, DCX exhibits high heterogeneity across different maturation stages of NBNs (*58*), and our observations at a single time point and limited number of neurons may not sufficiently depict the dynamic changes and outcomes of specific and overall populations of NBNs after functional silencing. Therefore, further exploration of methods to mark and track specific NBN populations, and investigating the impact of NBN silencing on synaptic plasticity and neural network disruption may provide evidence to explain the mechanisms of cognitive impairment. Moreover, our sequential approach to initial and reversal learning in our cohorts could be influenced by changing physiological traits of NBNs over time, possibly requiring further maturation to manifest a notable impact on behavior during the initial learning. However, consistency in initial learning is vital to assess reversal learning capabilities, and our study primarily underscores entire hippocampal functional changes due to NBN loss at the observed time points. Thus, the training timeline adjustments and the targeting of NBNs at specific developmental stages might reveal more about their functional shifts. Finally, since fMRI provides indirect insights into neural activity, the interactions and the causality between NBNs and other hippocampal cell types deserve additional investigation. Future studies should examine NBN effects across the central nervous system, as well as their age-related and stimuli-driven evolution.

## MATERIALS AND METHODS

### Experimental design

The primary objective of our study is to elucidate the role of NBNs in modulating the overall functionality of the hippocampus, with a particular focus on their contribution to cognitive flexibility. We will use the NBN-TeTX mouse model, wherein NBNs are to be noninvasively silenced. This model allows us to probe potential changes in neural activities and consequent behaviors.

1) NBN silencing using the NBN-TeTX model: By using the NBN-TeTX mouse model, we aim to noninvasively silence the NBNs, which will pave the way to uncover the inherent intricacies of NBN functionalities.

2) Behavioral assessment: We will monitor the behavioral control by NBNs during initial and reversal learning phases using the Morris water maze and operant learning tasks.

3) fMRI analysis: In adherence to our noninvasive approach, fMRI will be used to characterize shifts in hippocampal activation patterns during operant learning after NBN silencing. This analysis involves observing and comparing during both initial learning and reversal learning phases.

4) Cellular activity analysis in the DG: By using immunohistochemistry, we aim to analyze the activity patterns of key cellular components within the DG.

### Transgenic composition of NBN-TeTX mice

The NBN-TeTX triple-transgenic mouse model was constructed by combining two distinct transgenic systems: *Nes-CreERT2* system (*39*) and *TetO-TeTX* system (*41*). For mice bearing these three recombinant genes, TeTX expression can be specifically induced after tamoxifen injection, suppressing neurotransmitter release by cleaving VAMP2 located in synaptic vesicles, thereby noninvasively inhibiting NBN function in the NBN-TeTX mice. For detailed mechanism, see the Supplementary Materials.

### Genotyping

To ascertain the presence of the target recombinant gene in transgenic mice, genotypes were identified via polymerase chain reaction (PCR), using DNA extracted from mouse tails. Mice aged over 3 weeks had a 5-mm segment of their tails excised and subsequently placed in a lysis buffer mixture (100 mM tris, 5 mM EDTA, 200 mM NaCl, 0.5% Tween 20, pH 8.5) containing proteinase K (Takara Bio Inc., Otsu, Japan). This mixture was incubated at 55°C for at least 12 hours. The extracted DNA was then reconstituted in 50 μl of TE buffer and served as the PCR template. Detailed primer sequences, reaction solution compositions, and reaction conditions pertinent to the genotyping process are presented in table S1.

### Tamoxifen administration

Tamoxifen was used to induce *Cre* recombinase activity in *CreERT2*-expressing cells. It was combined with a solution of corn oil and 100% ethanol at a 9:1 ratio, resulting in a tamoxifen concentration of 30 mg/ml. The test tube, enveloped in aluminum foil, was incubated at 53°C for an hour with periodic agitation to ensure tamoxifen dissolution. Subsequent incubation at 37°C for a minimum of 30 min was conducted with intermittent shaking. This mixture was maintained at 37°C until administered to the mice. Over a span of 3 days, mice were intraperitoneally administered a daily dose of 180 mg/kg. To calibrate the dosage, mice were weighed daily during the administration period. Sterilization of the needle tip was achieved using 70% ethanol before and after each dosing. For hygiene purposes, a fresh tamoxifen mixture was prepared daily. To negate potential tamoxifen influence on experimental outcomes, all experimental mice (including both NBN-TeTX and control groups) were injected with tamoxifen between 14 and 16 weeks of age. Moreover, all behavioral studies were initiated 3 weeks after tamoxifen administration (i.e., when mice were between 17 and 19 weeks of age).

### BrdU injection

Concurrent with tamoxifen administration, BrdU (FUJIFILM, 027-15561) was introduced to assess any alteration in the quantity of surviving NBNs in NBN-TeTX mice 8 weeks subsequent to tamoxifen dosing. BrdU was prepared as a solution (10 mg/ml) in saline and was delivered intraperitoneally at 100 mg/kg across three sequential days. For the discernment and enumeration of matured NBNs, immunohistochemical staining using BrdU/NeuN markers was conducted.

### Retrovirus preparation and infection

The Moloney viral vector was generously donated by T. Nakashiba (*42*). All vector solutions were prepared at a concentration of 1 g/liter in TE buffer (pH 8.0). Retroviral injections commenced 3 days after tamoxifen administration. Under anesthesia, mice received an injection of the virus into the right DG via a mineral oil–filled glass micropipette attached to Nanoject II (Drummond) at coordinates [−2.06 mm anteroposterior (AP), 1.25 mm mediolateral (ML), −1.75 mm dorsoventral (DV)] and (−2.7 mm AP, 2.0 mm ML, −1.75 mm DV). At each location, 897 nl (69 nl, 13 injections) of the retrovirus was administered at a rate of 0.8 liter/min. After injection, the micropipette remained in situ for 3 min before extraction.

### Fixation, sectioning, and immunohistochemistry

Mice underwent transcardiac perfusion with phosphate-buffered saline (PBS) (137 mM NaCl, 8.1 mM Na_2_HPO_4_, 2.68 mM KCl, and 1.47 mM KH_2_PO_4_) followed by 4% paraformaldehyde (PFA). Brains, after perfusion, were fixed overnight at 4°C in 4% PFA and then underwent a gradient dehydration over 3 days in 10%, 20%, and 30% sucrose solutions. Subsequently, brains were embedded in O.C.T. Compound (Sakura, Tokyo, Japan) and sectioned into 40-μm slices using a cryostat (Thermo Fisher Scientific). Free-floating sections underwent immunofluorescence using standard protocols, after washing with tris-buffered saline (TBS; 137 mM NaCl, 2.68 mM KCl, 25 mM tris, pH 7.2).

A list of primary antibodies and their dilutions was as follows: GFP/mCherry: anti-GFP antibody (EPR14104) (1:2000, Abcam, catalog no. ab183734, RRID: AB_2732027) and Living Colors DsRed polyclonal antibody (1:200, Takara Bio, catalog no. 632496, RRID: AB_10013483); BrdU: anti-BrdU (1:200, Abcam, catalog no. ab6326, RRID: AB_305426); DCX: doublecortin antibody (C-18) (1:200, Santa Cruz Biotechnology, catalog no. sc-8066, RRID: AB_2088494); NeuN: anti-NeuN antibody, clone A60 (1:1000, Sigma-Aldrich, catalog no. MAB377, RRID: AB_2298772); GFAP: monoclonal anti-glial fibrillary acidic protein (GFAP) antibody produced in mouse (1:1000, Sigma-Aldrich, catalog no. G3893, RRID: AB_477010); SOX2: SOX2 antibody (N1C3) (1:200, GeneTex, catalog no. GTX101507, RRID: AB_2038021); PV: monoclonal anti-parvalbumin antibody produced in mouse (1:1000, Sigma-Aldrich, catalog no. P3088, RRID: AB_477329); GluR2/3: anti-glutamate receptors 2 and 3 (1:500, Millipore, catalog no. AB1506, RRID: AB_90710); c-Fos: c-Fos antibody (4) (1:200, Santa Cruz Biotechnology, catalog no. sc-52, RRID: AB_2106783) and goat polyclonal anti-c-Fos (1:200, Santa Cruz Biotechnology, catalog no. sc-52-G, RRID: AB_2629503); DAPI (4′,6-diamidino-2-phenylindole) (1:1000, Sigma-Aldrich, catalog no. D9542).

### Immunofluorescence image acquisition and processing

For GFP/mCherry costaining quantification at CA3-projecting synapses, images were captured using an oil immersion lens at ×63 magnification on a confocal microscope (TCS SP2, Leica, Germany). Four slices per mouse were used for the synaptic costaining count over the full DG span (*n* = 3 per group). Additional imaging of immunolabeled specimens was done using confocal laser scanning microscopes (FLUOVIEW FV3000, Olympus, Japan) with 20× objectives.

To estimate the total NBN count (BrdU^+^/NeuN^+^) in the hippocampal DG, systematic sampling was used. Three interval slices (each 40 μm thick) covering the entirety of the DG were selected for each genotype (at the 4-week characterization, *n* = 3; at the 8-week characterization, *n* = 5). Using a z-stack under a 40× objective, NBNs in both the left and right DG were counted for each selected slice. The cell count average from three sections per mouse was then multiplied by 60, based on the 2.4-mm standard length of the DG in mice, to approximate the total cell count across the entire DG.

In the morphological analysis of immature NBNs (DCX^+^/NeuN^−^) 4 weeks after tamoxifen injection, several sections with typical DG structure covering both dorsal and ventral parts were selected. We chose NBNs with identifiable, nonoverlapping cell bodies and typical dendritic structures for analysis. From each of the two groups, 20 neurons were randomly selected from each of three mice, and the data were pooled for statistical analysis with a total of 60 neurons per group.

For c-Fos–associated immunofluorescence staining, resting-state slices were procured from two mouse groups without any prior learning or training experiences. Following removal from the breeding room during light cycle, these mice were immediately perfused and fixed. Slices related to the Go and Nogo tasks came from mice perfused and fixed within 15 min after completion of the learning tasks. In each case, three male mice from both groups were chosen. For each condition, slices were sectioned at 40 μm thickness from hippocampal areas, illustrating typical dorsal and ventral DG structures. The c-Fos–expressing cells’ percentage in every slice was determined relative to the total target cells. Imaging involved z-stacks at a pixel resolution of 1024 × 1024.

Image processing was executed via Fluoview Software Module 3 and further analyzed with Fiji/ImageJ (*89**,* *90*), ensuring no selective adjustments or modifications to specific image segments.

### Selection of behavioral experimental subjects

In all the tests, mice that had the three previously mentioned genes made up the experimental group, while the control group consisted of mice that lacked one or both transgenes. Additionally, only male mice were used in both the NBN-TeTX and control groups. This decision was taken to eliminate potential discrepancies in BOLD signals and variations in behavioral outcomes that could arise from factors like brain size differences and variations in anxiety levels. Mice chosen for these behavioral tests were administered tamoxifen when they were between 14 and 16 weeks of age and were subsequently tested from weeks 17 to 19. All the behavioral trials were conducted during the light phase of the day. During the experiments, the experimenter was unaware of the mouse genotypes. All animal protocols and experiments were approved by the University of Tokyo’s Ethical Committee (B-18-03) and conducted according to its Guidelines for Animal Experimentation, ensuring adherence to the institutional Guide for Laboratory Animal Care and Use.

### Morris water maze

In an effort to understand the effects of neuronal functional inhibition on reversal learning capabilities, a training regimen spanning 10 days alongside three spatial exploration trials was carried out. These involved 13 NBN-TeTX mice and 11 control mice. The said spatial exploration trials took place on days 5, 10, and 13.

The time to reach the platform (latency) was calculated and used as a criterion of spatial memory to evaluate the mice’s learning. The first 5 days (days 1 to 5) were dedicated to acquisition learning. The platform was set in the NE quadrant, with learning sessions repeated six times daily. The first spatial exploration trial (P1) was initiated immediately after the fifth day of acquisition learning. Reversal learning was the focus of days 6 to 10. During this phase, the platform was repositioned to the SW quadrant, and mice were trained three times daily, with sequential randomization and 30 s between each training session. Spatial exploration trial P2 followed right after the 10th day’s reversal learning.

In total, three spatial exploration trials (P1, P2, and P3) were conducted. While P1 and P2 aimed to gauge memory retention across the respective learning stages, P3 (carried out after a 72-hour gap after day 10) assessed the establishment of long-term memory after the entire 10-day learning period. Figure S2B displays the swimming trajectories of two randomly selected individuals from both groups at P1, P2, and P3. All data from this experiment were collected and analyzed using the animal experiment analytical software, Smart 3.0 (by Panlab Harvard Apparatus). The detailed protocol can be found in the Supplementary Materials.

### Operant learning experiment

The operant learning experiment in this study used an operant learning apparatus that uses light stimulation to cue mice to lick water and has been used to measure the executive function of the mice accurately. Mice are light-cued while their heads are immobilized in this device, and water is given as a reward when a sensor near their mouths detects a tongue-extending lick in response to the light stimulus (Fig. 3C). We used this device to perform Go task and Nogo task to investigate the effects of silencing NBNs on executive function.

On the last day of the handling, the mice were restricted to a maximum daily water intake of 2000 μl until the end of the training. Following the handling, a 3-day reflex-to-light training was conducted. The light cue was constantly on and was briefly turned off when the mice actively licked and were rewarded with water (4 μl × two times).

Three weeks after tamoxifen injections, a 10-day Go task was performed. Again, the mice were restricted to a maximum daily water intake of 2000 μl on the last day of the handling until the end of the experiments. During the Go task, the mice were given a light cue of 2-s duration, and if the mice licked voluntarily during this 2 s, they were considered correct and given a water reward. Immediately after the Go task, a 10-day Nogo task was performed, during which the mouse was also given a 2-s light cue, was considered correct if it did not lick during this period, and was given water rewards immediately after the light cue.

However, the rules of the operant learning experiments performed in fMRI are slightly changed (Fig. 3A). First, each trial was set to 20 s due to the block design and given a light cue during the initial 2 s (0 to 2 s). Meanwhile, in the Nogo task, the rule was set to be considered correct only when the mouse actively licked within 2 s after the light cue went off to reduce the possibility of false correctness due to mouse inaction (omission) caused by the relatively cold environment and noise during the fMRI scan. That is, the water reward was given only when the subject actually comprehended the rule change and performed the correct licking behavior. The detailed protocol can be found in the Supplementary Materials.

### tb-fMRI analysis

#### Equipment and location

The tb-fMRI analysis was conducted at the Graduate School of Frontier Sciences, The University of Tokyo. A high-precision 14-T small horizontal bore animal scanner from Jastec, Japan was used, paired with a mouse head–adapted cryocoil and the ParaVision 5.0.1 software suite.

#### Sample

The subject pool consisted of six male NBN-TeTX mice and six control male mice, all of which had successfully completed the licking training.

#### Procedure

Mice were positioned with their custom resinous headplate (attached during previous surgeries) at the center of the cryocoil. The headplate installation and preprocessing steps before scanning were adopted from the work of T. N. A. Dinh (*45*). The water tube’s position was adjusted to facilitate easy reward access for the mice. To ascertain the correct positioning of the mice, tripilot images were taken. tb-fMRI scanning lasted for 6 min and 10 s, with a preliminary 10-s window to stabilize the fMRI signal.

The specific parameters for the fMRI scans were as follows: SE-EPI: repetition time (TR) = 2000 ms, echo time (TE) = 18 ms, flip angle = 90°, matrix size = 64 × 64, field of view (FOV) = 17.2 × 17.2 mm^2^, 20 slices, interleaving sequence, 0.7 mm slice thickness, no gap, BW = 300 kHz. T2-weighted anatomical images: TR = 800 ms, TE = 24 ms, flip angle = 90°, matrix size = 256 × 256, FOV = 17.2 × 17.2 mm^2^, 20 slices, interleaved sequence, 0.7 mm slice thickness, no gap.

#### tb-fMRI data preprocessing

tb-fMRI data preprocessing was mainly processed using SPM12, and the downsample Australian Mouse Brain Mapping Consortium (AMBMC) template (*91*) was used for coregister, mask creation, and result mapping (10 times and reorient of the coordinates occupied the midpoint of the anterior commissure; dimensions: 68 × 131 × 50 voxels, voxel size: 0.15 × 0.15 × 0.15 mm).

The first-level analysis was conducted using a general linear model (GLM), with the inclusion of six regressors representing the transitional and rotational motion parameters. Group-level results were derived using a one-sample *t* test with a significance level set at cluster level probability false discovery rate (pFDR) < 0.05 and cluster size threshold = 15. For discerning statistically significant activations, a threshold was set at pFDR < 0.05 with a cluster size threshold of 15 voxels. The detailed protocol can be found in the Supplementary Materials.

### rs-fMRI analysis

#### Location and equipment

The rs-fMRI study was carried out at the Okinawa Institute of Science and Technology Graduate University. MRI data collection used an 11.7-T small horizontal bore animal scanner (BioSpec 117/11, Bruker, Ettlingen, Germany), along with a mouse head–adapted cryocoil and ParaVision 6.0.1 software.

Twenty-eight mice participated in this study, with 14 being NBN-TeTX mice and the remaining 14 as controls. Mice were anesthetized using isoflurane (3% for initiation and 1.5% during MRI). Each mouse was placed in a prone position on a custom MRI bed fitted with a bite bar and gas mask. Core body temperature was maintained at 37.0 ± 1.0°C via a water-circulating pad, with monitoring facilitated by an MRI-compatible rectal temperature probe (Model 1025, SA Instruments). Tripilot images were taken to ensure correct mouse positioning in relation to the coil and magnet’s isocenter. The rs-fMRI ran for 6 min, where the imaging parameters included the following: SE-EPI: TR = 1800 ms, TE = 18 ms, flip angle = 90°, matrix size = 96 × 96, FOV = 20 × 20 mm^2^, 22 slices, slice thickness of 0.5 mm, no slice gap, BW = 500 kHz, and 200 total volumes. T2-weighted anatomical images: TR = 3374 ms, TE = 30 ms, flip angle = 90°, matrix size = 256 × 256, FOV = 20 × 20 mm^2^, 34 slices, slice thickness of 0.5 mm, no gap, with two averages.

#### rs-fMRI data preprocessing

rs-fMRI data preprocessing was mainly processed using SPM12 and CONN toolbox (*92*), and 3D Slicer was used for the segmentation and definition of the ROIs (*93*). The detailed protocol can be found in the Supplementary Materials.

### Statistical analysis

Statistical analyses for immunofluorescence staining and behavioral experiments were conducted using either unpaired *t* test or two-way repeated-measures analysis of variance (ANOVA) with Bonferroni post hoc test, as appropriate. GraphPad Prism 9.4.0 (673) for Windows was the primary tool for these analyses. For fMRI correlation analyses, the software’s built-in algorithm was used. Significance was set at *P < 0.05. Unless otherwise specified, error bars represent mean ± SEM.
